# Anticipatory action planning in blind and sighted individuals

**DOI:** 10.1038/srep44617

**Published:** 2017-03-17

**Authors:** Andrea Cavallo, Caterina Ansuini, Monica Gori, Carla Tinti, Alessia Tonelli, Cristina Becchio

**Affiliations:** 1 Department of Psychology, University of Turin, Italy; 2 Cognition, Motion and Neuroscience Unit, Fondazione Istituto Italiano di Tecnologia, Genova, Italy; 3 Unit for Visually Impaired People, Fondazione Istituto Italiano di Tecnologia, Genova, Italy

## Abstract

Several studies on visually guided reach-to-grasp movements have documented that how objects are grasped differs depending on the actions one intends to perform subsequently. However, no previous study has examined whether this differential grasping may also occur without visual input. In this study, we used motion capture technology to investigate the influence of visual feedback and prior visual experience on the modulation of kinematics by intention in sighted (in both full-vision and no-vision conditions), early-blind and late-blind participants. Results provide evidence of modulation of kinematics by intention to a similar degree under both full-vision and no-vision conditions. Moreover, they demonstrate that prior visual experience has little impact on the tailoring of grasping movements to intention. This suggests that sequential action planning does not depend on visual input, and may instead be ascribed to the function of multisensory-motor cortical network that operates and develops not only in light, but also in darkness.

Human beings and other primates are capable of reaching towards, and grasping objects with great precision^1^,^2^. The control of this skilled behaviour has been attributed to the function of a specialized *visuomotor* cortical network, which transforms the visual attributes of the object to be grasped into an appropriate hand configuration^3^.

Although vision offers distinctive input to this network, several observations indicate that the lack of visual experience may have only limited effects on skilled hand functions^4^,^5^,^6^,^7^,^8^. Early visual deprivation, for example, does not prevent development of the ability to point at targets^7^,^9^,^10^,^11^ and accurately reach towards and grasp objects^5^ (although it may impair spatial metrics^12^ and the ability to judge orientation^13^). Moreover, there is evidence that sighted individuals who are blindfolded can use proprioceptive feedback alone to initiate and guide rapid on-line adjustments of hand orientation during reaching movements toward a stationary target^10^.

These studies challenge the view that visual input is indispensable for the appropriate motor control of *single-step* reach-to-grasp movements. Motor behaviour in daily life, however, demands much more complex control than that required for control and guidance of isolated movements, including the ability to chain together distinct motor acts to achieve an overarching goal. If, while in a restaurant, for example, we grasp a bottle, we commonly do so as part of a more complex, meaningful action sequence, such as pouring a glass of wine, or passing the bottle to another person. When performing a sequential action of this kind, the initial movements differ substantially as a function of the actions actors *intend* to perform subsequently^14^,^15^,^16^,^17^,^18^,^19^,^20^,^21^,^22^,^23^,^24^. For example, if the goal were to pour a glass of wine, we would grasp the wine bottle closer to the bottom in order to facilitate a pouring action. Moreover, in preparation of object rotation, reaching movements would typically be longer, and the fingers more extended compared to when the intent is to pass the bottle to another guest^15^,^22^. On the other hand, if the goal were to chill the wine, we would need to hold the bottle closer to the top to facilitate the insertion of the bottle into an ice bucket^25^.

This *modulation of kinematics by intention*, which is thought to reflect anticipatory action planning, is well documented for visually guided reach-to-grasp movements^26^,^27^. To the best of our knowledge, however, no previous study has investigated whether it may also occur without visual input. Sighted individuals use vision and task-specific eye movements to support sequential movement planning and control^28^,^29^,^30^,^31^. In visually guided sequential movements, gaze is typically directed to successive contact locations, arriving before the hand, and departing around the time the sub-goal is completed^28^,^29^,^31^,^32^,^33^,^34^. These proactive gaze movements have been shown to be associated with learning to predictively link successive action phase^35^,^36^. Thus, the question arises whether such learning can also occur in individuals with limited or no visual experience. Moreover, there is no information in the literature regarding the role of visual input during execution of sequential actions in sighted individuals. Put differently: is the visual feedback one gains through proactive gaze movements indispensable for tailoring one movement to the next?

In this study, we sought to address these questions by examining the role of vision in the control and guidance of grasp-to-pour, grasp-to-place, and grasp-to-pass movements (Fig. 1). These three actions were chosen as they are well known to differ according to the intended goal when performed under visual guidance^15^,^22^. First, to determine the influence of *visual feedback* (i.e., the availability of visual input during movement performance) on differential grasping, we compared the execution of movements performed by sighted participants under full-vision and no-vision (blindfolded) conditions. Next, to quantify the influence of prior *visual experience* (i.e., the experience of visual input during ontogenesis), we compared grasping kinematics of blindfolded sighted participants with those of early-blind participants, who have never experienced visual feedback and who have no memory of motor performance under visual guidance, and late-blind participants, who lost their sight after the age of 6.

## Results

Below we report the results for the analyses of the influence of visual feedback and visual experience separately. Mean and standard errors are available for each condition and each group as Supplementary Table S1 and Supplementary Table S2.

### Influence of visual feedback in sighted participants

The MANOVA on *peak* and *time-to-peak* variables revealed a main effect of both ‘visual input’ [F_1,12_ = 45.518; p < 0.05] and ‘intention’ [F_22,30_ = 4.393; p < 0.001]. The two-way ‘visual input’ by ‘intention’ interaction also reached statistical significance [F_22,30_ = 2.631; p < 0.01]. Repeated measures ANOVAs revealed a main effect of ‘visual input’ on *MD, PV, TPV, PA, TPA, PD, TPD, PG*, and *TPG* [F_1,12_ ranging from 5.667 to 98.880; p_s_ < 0.05; 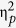 = ranging from 0.321 to 0.892]. As depicted in Fig. 2, inspection of the main effects of ‘visual input’ revealed that *MD* was longer, *PV* was lower, and occurred earlier in the no-vision than in the full-vision condition (p_s_ < 0.001). In addition, both acceleration and deceleration peaks were smaller (p_s_ < 0.001) and occurred earlier in the no-vision than in the full-vision condition (p_s_ < 0.02). The kinematic pattern of the finger grip was also modified in absence of visual input, with *PG* being larger (p < 0.05) and occurring earlier (p < 0.001) in the no-vision than in the full-vision condition.

The repeated measures ANOVAs also yielded a main effect of ‘intention’ on *MD, PV, PA*, and *PD* [F_2,24_ ranging from 13.514 to 32.592; p_s_ < 0.001; 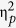
ranging from 0.530 to 0.731]. Post hoc pairwise comparisons revealed that movement duration was longer for grasp-to-pour compared to grasp-to-pass (p = 0.009) and grasp-to-place movements (p = 0.001). This lengthening of movement duration was reflected in lower velocity, acceleration, and deceleration peaks for grasp-to-pour movements in comparison to both the grasp-to-pass and grasp-to-place movements. *PV* was indeed lower for grasp-to-pour movements than for both the grasp-to-pass (p = 0.005) and grasp-to-place movements (p < 0.001). *PV* was also lower for grasp-to-pass than for the grasp-to-place movements (p = 0.005). Similarly, *PA* was lower for grasp-to-pour than for both the grasp-to-pass (p = 0.005) and grasp-to-place movements (p < 0.001). Again, *PA* was also lower for grasp-to-pass than for grasp-to-place movements (p = 0.035). Finally, *PD* was lower for grasp-to-pour than for both grasp-to-pass (p = 0.014) and grasp-to-place movements (p < 0.001).

Surprisingly, the ‘visual input’ by ‘intention’ interaction was significant only for *TPH* [F_2,24_ = 16.844; p < 0.001; 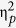 = 0.584]. Figure 3 shows post hoc comparisons results for the inspection of this interaction. As evident, the visual input did not influence *TPH* for grasp-to-pour and grasp-to-pass movements (p_s_ = 0.314 and 0.672 respectively). In contrast, for grasp-to-place movements, *TPH* occurred significantly earlier in the no-vision than in the full-vision condition (p = 0.007).

The 2 × 3 × 10 MANOVA on *resampled* variables yielded main effects of ‘visual input’ [F_3,10_ = 86.079; p < 0.001], ‘intention’ [F_6,46_ = 8.974; p < 0.001], and ‘time’ [F_27,324_ = 21.800; p < 0.001]. These main effects were qualified by two-way interactions between ‘visual input’ by ‘intention’ [F_6,46_ = 2.310; p = 0.049], ‘visual input’ by ‘time’ [F_27,324_ = 9.645; p < 0.001], and ‘intention’ by ‘time’ [F_54,648_ = 9.285; p < 0.001]. Importantly, these interactions were, in turn, moderated by a three-way ‘visual input’ by ‘intention’ by ‘time’ interaction [F_54,648_ = 2.790; p < 0.001]. When considering each dependent measure separately, ANOVA on *RV* revealed a significant ‘visual input’ by ‘time’ interaction (F_9,108_ = 38.797, p < 0.001, 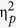 = 0.764). As shown in Fig. 4, *RV* was lower in the no-vision condition compared to the full-vision condition, from 40% of movement duration to the time of contact (p_s_ ranging from 0.000 to 0.001). The two-way interaction ‘intention’ by ‘time’ on *RV* was also significant [F_18,216_ = 7.689, p < 0.001, 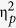 = 0.391], reflecting higher *RV* for grasp-to-place compared to both grasp-to-pass (from 50% to 60% of movement time, p_s_ = 0.016 and 0.003 respectively) and grasp-to-pour movements (from 20% to 70% of movement time, p_s_ ranging from <0.001 to 0.012). Grasp-to-pass movements were also faster than movements intended to pour (from 40% to 50% of movement time, p_s_ = 0.027 and <0.001 respectively). However, the central manipulation of visual input did not influence ‘intention’ [F_2,24_ = 1.175, p = 0.326, 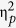 = 0.089], indicating that tailoring of *RV* to intention was not moderated by visual feedback. The three-way ‘visual input’ by ‘intention’ by ‘time’ interaction was also not significant [F_18,216_ = 1.187, p = 0.273, 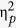 = 0.090].

As depicted in Fig. 4, the ANOVA on *RG* also revealed a significant two-way interaction ‘visual input’ by ‘time’ [F_9,108_ = 19.358, p < 0.001, 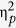 = 0.617], due to an increase in *RG* between 30% and 70% of movement duration in the no-vision condition compared to the full-vision condition (p_s_ ranging from 0.000 to 0.002). There was also a significant ‘intention’ by ‘time’ interaction [F_18,216_ = 3.379, p < 0.001, 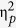 = 0.220], resulting from wider grip aperture for grasp-to-pass than grasp-to-place movements from 40% to 70% of movement duration (p_s_ ranging from 0.021 to 0.038), and for grasp-to-pour than grasp-to-place movements at 70%, 90%, and 100% of movement duration (p_s_ ranging from 0.018 to 0.042). Again, ‘visual input’ did not interact with ‘intention’ [F_2,24_ = 1.000, p = 0.383, 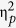 = 0.077], indicating that intention modulation of *RG* did not differ between full-vision and no-vision conditions.

Only for *RH*, the ANOVA revealed a significant three-way interaction [F_18,216_ = 8.300; p < 0.001; 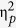 = 0.409]. Figure 5 visualizes results from post hoc pairwise comparisons for this interaction. From 10% to 40% of movement duration, for all three intentions, *RH* was higher in the no-vision condition than in the full-vision condition (p_s_ ranging from 0.000 to 0.029). For grasp-to-pass and grasp-to-pour movements, this pattern was consistent over time, reaching a statistical significance from 90% to 100% for grasp-to-pass movements. For grasp-to-place movements, it reversed with wrist height being greater in the full-vision than in the no-vision condition from 80% to 100% of the movement time (p_s_ ranging from 0.012 to 0.038).

In sum, these findings demonstrate that, under both full-vision and no-vision conditions, the initial reach-to-grasp movement is planned as part of a larger action sequence, which includes the actor’s intended future actions. Only for wrist height (*TPH* and *RH*) tailoring to onward action was influenced by visual feedback, with peak of wrist height occurring earlier and being significantly lower for blindfolded grasp-to-place movements.

### Influence of visual experience in early-blind, late-blind, and blindfolded sighted participants

The MANOVA on *peak* and *time-to-peak* variables revealed a main effect ‘intention’ [F_22,11_ = 7.044; p = 0.001]. Neither the main effect of ‘visual experience’ nor the ‘visual experience’ by ‘intention’ interaction reached the statistical significance. When considering each dependent measure separately, ANOVAs revealed the main effect of ‘intention’ on *MD, PV, TPV, PA, TPA, PD, TPD, PH, TPH, PG* and *TPG* [F_2,64_ ranging from 4.056 to 28.402; p_s_ < 0.05; 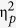 ranging from 0.111 to 0.470]. Post hoc pairwise comparisons showed that, in all three groups of participants, *MD* was prolonged for grasp-to-pour compared to both grasp-to-place and grasp-to-pass movements (p_s_ < 0.001). Conversely, *PV* was higher for grasp-to-place compared to both grasp-to-pass (p < 0.001) and grasp-to-pour movements (p < 0.001). *TPV* occurred earlier for grasp-to-pour compared to both grasp-to-place (p = 0.003) and grasp-to-pass movements (p = 0.027). *PA* was higher for grasp-to-place compared to both grasp-to-pass (p = 0.005) and grasp-to-pour movements (p = 0.002), and occurred later in the grasp-to-place movement compared to grasp-to-pour movements (p = 0.03). Similarly, *PD* was greater for grasp-to-place compared to both grasp-to-pass (p = 0.032) and grasp-to-pour movements (p < 0.001), and occurred later in grasp-to-pass compared to grasp-to-pour movements (p = 0.026). *PH* was also higher for grasp-to-place compared to grasp-to-pass movements (p < 0.001). Moreover, *TPH* occurred later in grasp-to-place compared to grasp-to-pour movements (p = 0.009). Finally, *PG* was greater, and *TPG* was reached later, for grasp-to-pass compared to both grasp-to-place and grasp-to-pour movements (p_s_ ranging from <0.001 to 0.047).

The 3 × 3 × 10 MANOVA on *resampled* variables yielded main effects of ‘intention’ [F_6,126_ = 8.112; p < 0.001], and ‘time’ [F_27,864_ = 54.185; p < 0.001]. There was no main effect of ‘visual experience’ [F_6,62_ = 1.367; p = 0.242]; however, there was a significant ‘visual experience’ by ‘time’ interaction [F_54,864_ = 1.818; p < 0.001]. The interaction ‘intention’ by ‘time’ was also significant. [F_54,1728_ = 8.990; p < 0.001]. Critically, the three-way interaction ‘visual experience’ by ‘intention’ by ‘time’ was also significant [F_108,1728_ = 1.351; p < 0.05], indicating that intentional modulation differed in the three groups, over time. When considering each dependent measure separately, ANOVAs revealed a significant three-way interaction for *RV* [F_36,576_ = 1.986; p < 0.001; 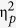 = 0.110]. As shown in Fig. 6, post hoc pairwise comparisons revealed no influence of visual experience over time for grasp-to-pass movements (p_s_ ranging from 0.076 to 1.000). In contrast, for grasp-to-pour movements, *RV* of early-blind participants at 30% of movement time was higher compared to that of blindfolded sighted participants (p = 0.031). For grasp-to-place movements, *RV* of early-blind participants at 40% of movement time was also higher compared to that both of late-blind (p = 0.019) and blindfolded, normally sighted participants (p = 0.027) (Fig. 6).

Three-way interactions on *RH* and *RG* failed to reach significance (p_*s*_ = 0.176 and 0.439 respectively). However, the two-way interactions ‘visual experience’ by ‘time’ and ‘intention’ by ‘time’ were significant on both *RH* [F_18,288_ = 2.332, p < 0.01, 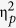 = 0.127 and F_18,576_ = 11.618, p < 0.001, 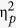 = 0.266, respectively) and *RG* (F_18,288_ = 2.150, p < 0.01, 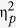 = 0.118 and F_18,576_ = 3.436, p < 0.001, 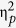 = 0.097, respectively]. As visible in Fig. 7, for what concerns the ‘visual experience’ by ‘time’ interaction found on RG, post hoc pairwise comparisons revealed that at contact (100% of movement time), grip aperture was larger, and thus presumably less stable^37^ for blindfolded sighted participants than for early-blind participants (p = 0.026). *RG* was also larger for grasp-to-pass compared to grasp-to-place movements from 70% to 100% of movement time (p_s_ ranging from 0.001 to 0.049), and for grasp-to-pass compared to grasp-to-pour movements at 70% and 80% of movement time (p_s_ = 0.048 and 0.007, respectively) (Fig. 7).

Finally, for *RH*, the exploration of the ‘visual experience’ by ‘time’ interaction failed to reveal any modulation by visual experience, over time. The results showed, however, a significant influence of time on intention modulation, with the wrist height being higher for grasp-to-place in comparison to both grasp-to-pass movements from 30% to 100% of movement time (p_s_ ranging from <0.001 to 0.012) and grasp-to-pour movements from 50% to 100% of movement time (p_s_ ranging from 0.006 to 0.047). *RH* was also higher for grasp-to-pour than for grasp-to-pass movements at 20% and 30% of movement duration (p_s_ = 0.026 and 0.039, respectively).

In sum, these results indicate that only the modulation of *wrist velocity* by intention was influenced by visual experience, with *RV* being higher early in the movement for grasp-to-pour and grasp-to-place movements in early-blind participants in comparison to both late-blind participants and blindfolded sighted participants.

## Discussion

How objects are grasped is not only determined by the immediate task demands (e.g., the perceived size and orientation of the object), but also differs depending on what one plans to do with the objects. Such differential grasping has been demonstrated in a wide range of sequential tasks performed under visual guidance. This suggests that sighted participants plan movements as a function of the intended goal of the larger action sequence in which they occur^14^,^15^,^16^,^17^,^18^,^19^,^20^,^21^,^22^,^38^. In the present study, we investigated for the first time whether (and to what extent) *visual feedback* and *visual experience* are necessary for the intentional patterning of grasping movements.

### Influence of visual feedback

Several times each day, we perform reach-to-grasp movements under conditions of full visual feedback; thus, it is unsurprising that a visual deprivation applied to subjects who normally use vision affects movement parametrization^39^. In line with this, we found that when vision was occluded in normally sighted participants, overall movement duration was increased. Acceleration and velocity peaks of the wrist were lower, and occurred earlier in time. Moreover, and in agreement with previous reports on single-step movements^40^,^41^,^42^, maximum grip aperture was larger, and occurred earlier in time for no-vision grasps.

Nevertheless, differential grasping as a function of intention was largely uninfluenced by visual feedback. Movement duration was prolonged for grasp-to-pour compared both to grasp-to-place and grasp-to-pass movements. Consistent with previous literature, velocity and acceleration peaks were also lower for grasp-to-pour in comparison to grasp-to-place and grasp-to-pass movements, and for grasp-to-pass in comparison grasp-to-place movements^22^. These effects were observed under both full-vision and no-vision conditions. Only for wrist height, kinematic analyses revealed an influence of visual input on the intentional patterning of grasping. Specifically, for grasp-to-place movements, wrist height peaked earlier, and was lower in the final part of the movement when vision was occluded. This pattern suggests that, in the absence of visual feedback, participants grasped the bottle lower in order ensure a larger margin of ‘safety’ for grasp-to-place movements. When grasped higher (above the shoulders of the bottle), the bottle may indeed be more easily missed or pushed over. An alternative, though not mutually exclusive, explanation is that participants adopted a different motor control strategy when grasping in the dark. Specifically, it is possible that participants adjusted grasp height in order to facilitate dropping of the bottle into the box, i.e., they grasped the bottle lower so that, during second phase of the movement, wrist/hand contacted the outer edge of the box, providing a reference point for dropping. At the time of contact with the object, wrist height for grasp-to-place movement was indeed 146 ± 31 mm. As the box was 125 mm height, this implies that a good proportion of participants grasped the bottle lower than the height of the box.

Overall, these findings indicate that visual feedback is not indispensable for the tailoring of grasping movements to intended actions. Sighted participants do not need visual feedback to predictively link the first grasping movement to the goal of the larger action sequence in which it occurs.

### Influence of visual experience

Previous studies have shown that even when deprived of visual feedback from very early in life, blind individuals still generate straight hand trajectories^6^, and accurately scale grip aperture to the size of the unseen object^5^, suggesting that the development of the patterning of the basic reach-to-grasp movement is largely independent from vision. However, the simple movements favoured in these experiments represent little more than elements of everyday action. Thus, the question remained whether the development of more complex forms of grasping control, such as those involved in sequential action planning, depends on visual experience.

Our results document for the first time that differential grasping as a function of intention is largely uninfluenced by prior visual experience. Analysis of kinematic landmarks, such as peaks and their times of occurrence, revealed no differences in the execution of movements performed by early-blind, late-blind, and blindfolded sighted participants. The evolution of kinematics over the whole movement duration was also strikingly similar across the three groups. Velocity and acceleration profiles overlapped, except that the wrist velocity of grasp-to-place movements was higher in early-blind participants than in late-blind and blindfolded sighted participants at 40% of normalized movement duration. Similarly, for grasp-to-pour, early in the movement (30% of normalized movement duration) the wrist velocity of early-blind participants was higher compared to that of blindfolded sighted participants.

A faster approach of the hand at these stages is compatible with more ‘confidence’ in performing the movement (see also ref. 5), and may reflect a ‘motor calibration’ effect^43^, possibly related to the influence of the age of blindness on the plastic reorganization of grasping functions^13^,^44^. The functionality of this pattern, however, remains to be determined. Do early-blind individuals show a more functional movement in comparison to late-blind and blindfolded sighted participants? As this study was not designed to test the efficiency of movement, this comparison cannot be made directly. However, indirect evidence can be obtained by comparing the jerk of the onward phase across groups. Jerk measures the rate of change of acceleration over a given time period; increased jerk suggests adjustments in the movement kinematic profile, and can therefore be used as an inverse measure of planning efficiency (for a similar approach see ref. 43). If changes in the reach-to-grasp phase were linked to changes in the efficiency of the action, we would expect jerk of the onward action to be reduced for early-blind individuals in comparison to both late-blind and blindfolded sighted participants. When comparing jerk of onward place and pour movements, however, we found no difference between groups (p = 0.539). Thus, our results provide no evidence that increased wrist velocity in early-blind individuals reflects a more efficient overarching motor plan.

### Multimodal control of grasping?

Recent reports indicate that, in sighted individuals, tailoring to onward action changes with practice, with an increase in velocity and decrease in the amount of time spent decelerating during the reach phase^43^. These changes have been interpreted as the result of the movement plan becoming more efficient with motor practice. In light of these results, it may seem surprising that visual feedback and visual experience exerted so little influence on the intentional modulation of grasping. After all, blind subjects are well practiced in using non-visual input signals to guide their arm and hand movements, whereas sighted subjects would intuitively appear to have little experience with the non-visual control of actions^45^,^46^,^47^,^48^,^49^,^50^. How, then, is it possible that sighted individuals used non-visual inputs to guide their performance as efficiently as late-blind participants with several years’ worth of experience in moving in the dark? The explanation for this apparent paradox may lie in the cortical organization of grasping. Findings from functional neuroimaging studies indicate that brain areas previously reported for visually guided hand movements – including the superior parietal cortex and the premotor cortex – are also activated by kinaesthetically guided hand movements^51^. Moreover, activations in areas of the cortical grasping network overlap between blind and sighted individuals^44^. For example, activity enhancement in the superior parietal cortex and the anterior parietal sulcus – an area that transforms sensory input into motor commands, and continuously updates the ongoing movement with respect to the aspired movement goal^52^ – has been reported in both congenitally blind and sighted individuals performing non-visually guided hand movements^51^. These findings have led to the proposal of a multimodal cortical network of hand movement control, accessible for both visually deprived and sighted individuals^44^,^53^. The similarities in grasping patterns across visual and non-visual tasks within sighted participants, and between early-blind, late-blind, and sighted participants in our study speak in favour of this hypothesis, suggesting that tailoring to onward action is largely independent from vision.

Other brain regions, including the pre-supplementary motor area, however, have been found to be selectively recruited by sighted samples, in comparison to congenitally blind samples^51^. The pre-supplementary motor area is proposed to implement higher order aspects of motor control involved in the updating of motor plans and control of complex movement sequences^54^. Future studies investigating action planning should therefore remain open to the possibility of differences in cortical activation patterns as a function of visual experience. Linking differential (and common) brain responses with differential (and common) movement profiles in sighted, early-blind, and late-blind participants is another important target for future research, combining kinematics and neuroimaging techniques.

### Conclusions

It was already well known that the way objects are grasped depends on the performer’s intention^26^. The novel finding here is that these effects are largely independent of *visual feedback* and *visual experience*. Our results provide evidence of modulation of kinematics by intention to a similar degree under both full-vision and no-vision conditions. Moreover, they demonstrate that exposure to visual input during ontogenesis has little impact on the tailoring of grasping movements to intention. These findings have important implications for models of anticipatory control, as they suggest that kinematic modulation by intention does not depend on visual input, and may instead be ascribed to the function of multisensory-motor cortical network – a network that operates and develops not only in light, but also in darkness.

## Materials and Methods

### Participants

Three groups of right-handed participants took part in this experiment: early-blind participants, late-blind participants, and sighted participants. The early-blind group consisted of 11 participants (five females; mean age: 40.27; range: 25–56). The late-blind group consisted of 11 participants (four females; mean age: 36.36; range: 22–66). The sighted group consisted of 13 sighted participants (six females; mean age: 33.77; range: 24–50). Details about age, pathology, and residual vision of the early-blind and late-blind participants are reported in Table 1. None of the sighted participants had a history of neurological problems, and they all had normal or corrected-to-normal vision. Written informed consent was obtained from each participant. The research was approved by a local ethical committee (ASL 3 Genovese) and was carried out in accordance with the principles of the revised Helsinki Declaration^55^.

### Stimuli and procedure

**Figure 1:**
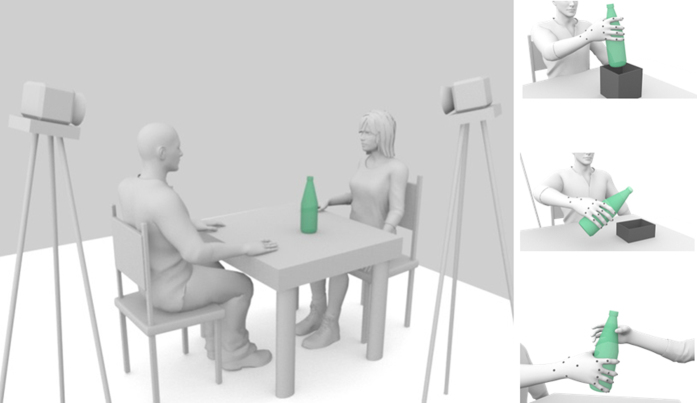
Schematic representation of the experimental task. Early-blind, late-blind, and sighted participants were instructed to reach and grasp the bottle with the intent to place it in a box (grasp-to-place), pour its content into a container (grasp-to-pour), or pass it to a co-experimenter (grasp-to-pass). Each participant was outfitted with 20 lightweight retro-reflective hemispheric markers that were tracked by near-infrared cameras.

**Figure 2:**
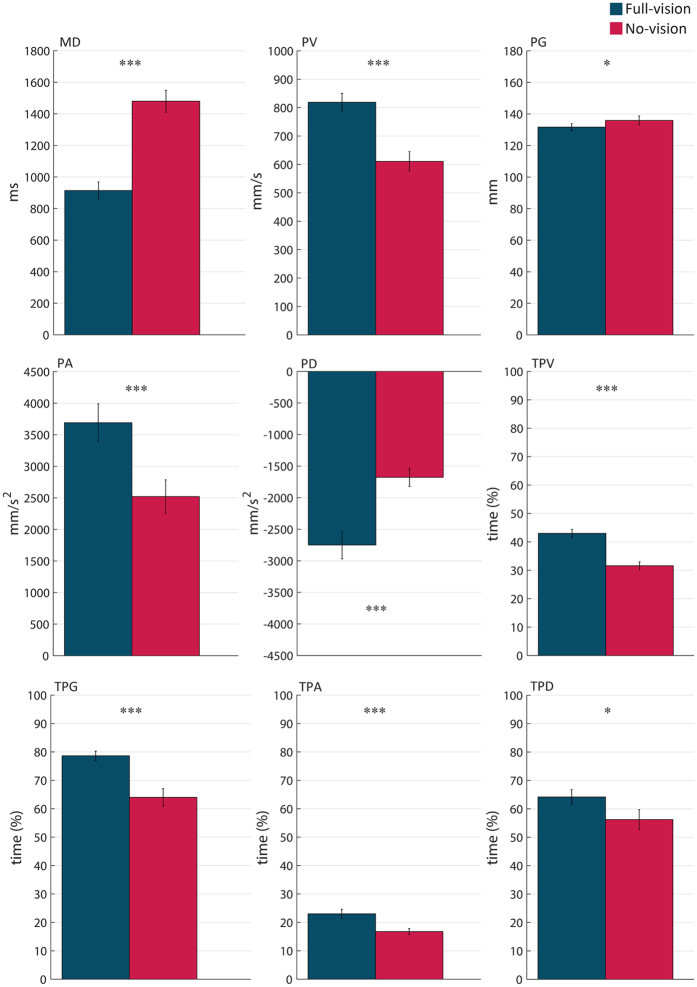
Main effect of ‘visual input’ in sighted participants. Histograms represent average values across participants for movement duration (*MD)*, peak velocity (*PV)*, peak grip aperture (*PG*), peak acceleration (*PA*), peak deceleration (*PD*), time to peak velocity (*TPV*), time to peak grip aperture (*TPG*), time to peak acceleration (*TPA*), and time to peak deceleration (*TPD*) under full-vision and no-vision conditions. Error bars indicate standard errors of the mean. One and three asterisks stand for p < 0.05 and p < 0.001, respectively.

**Figure 3:**
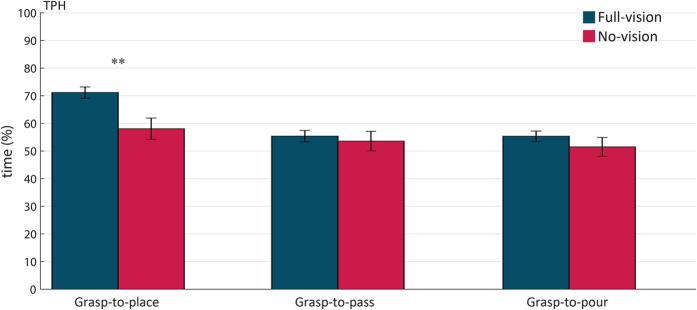
Interaction ‘visual input’ by ‘intention’ for time to peak height (TPH) in sighted participants. *TPH* occurred significantly earlier in the no-vision than in the full-vision condition for grasp-to-place but not for grasp-to-pass and grasp-to-pour movements. Error bars indicate standard errors of the mean. Two asterisks stand for p < 0.01.

**Figure 4:**
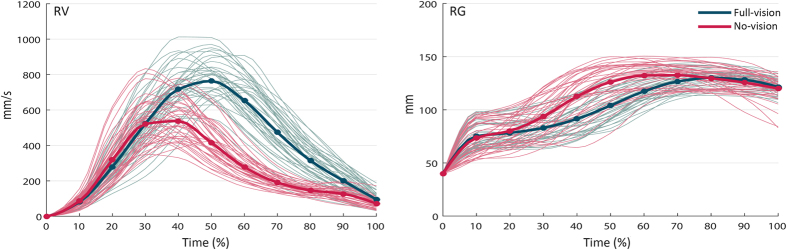
Interaction ‘Visual input’ by ‘time’ for resampled velocity (RV) and grip aperture (RG) in sighted participants. *RV* was lower in the no-vision condition than in the full-vision condition from 40% to 100% of movement time (*left panel*). *RG* was greater in the no-vision than in the full-vision condition from 30% and 70% of movement time (*right panel*). The bold lines represent group means; the light lines represent individual participants.

**Figure 5:**
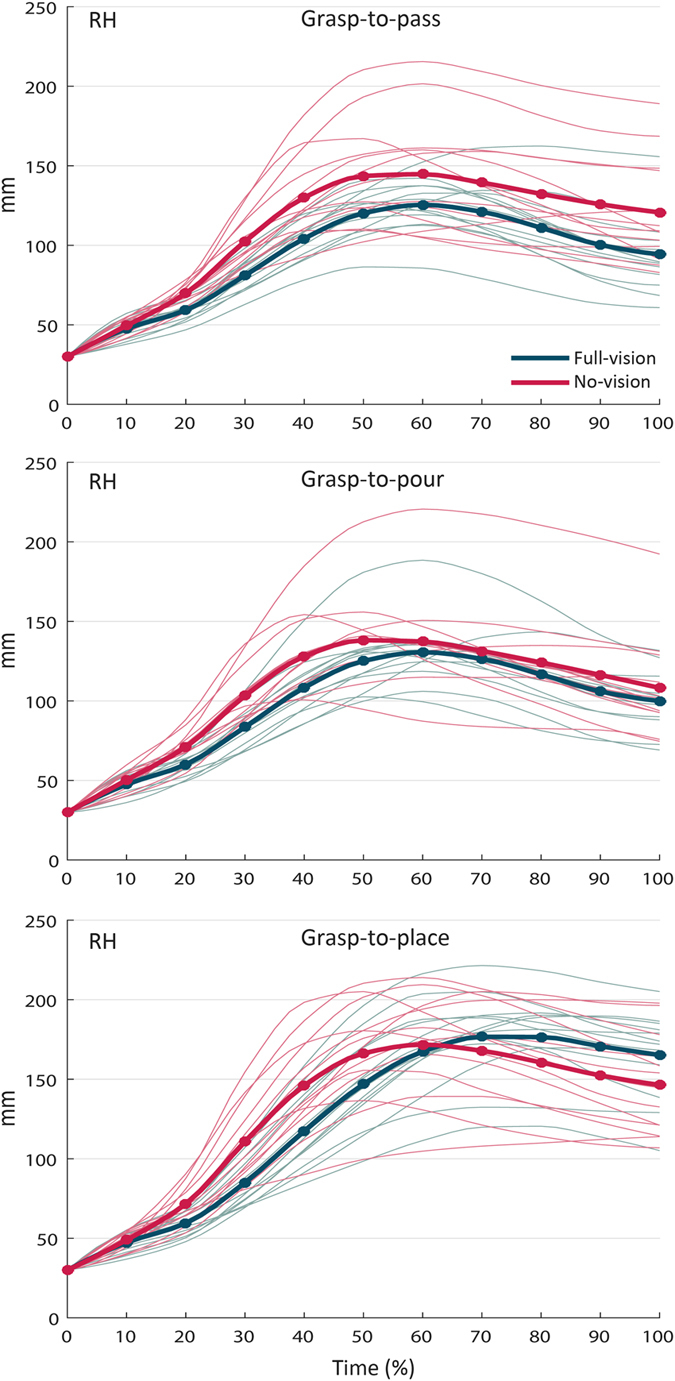
Interaction ‘visual input’ by ‘intention’ by ‘time’ for resampled wrist height (RH) in sighted participants. For all three intentions, *RH* was higher in the no-vision condition than in the full-vision condition from 10% to 40% of movement time. For grasp-to-pass and grasp-to-pour movements this pattern was consistent over time, reaching a statistical significance from 90% to 100% for grasp-to-pass movements. For grasp-to-place movements, it reversed with wrist height being greater in the full-vision than in the no-vision condition from 80% to 100% of the movement time. The bold lines represent group means; the light lines represent individual participants.

**Figure 6:**
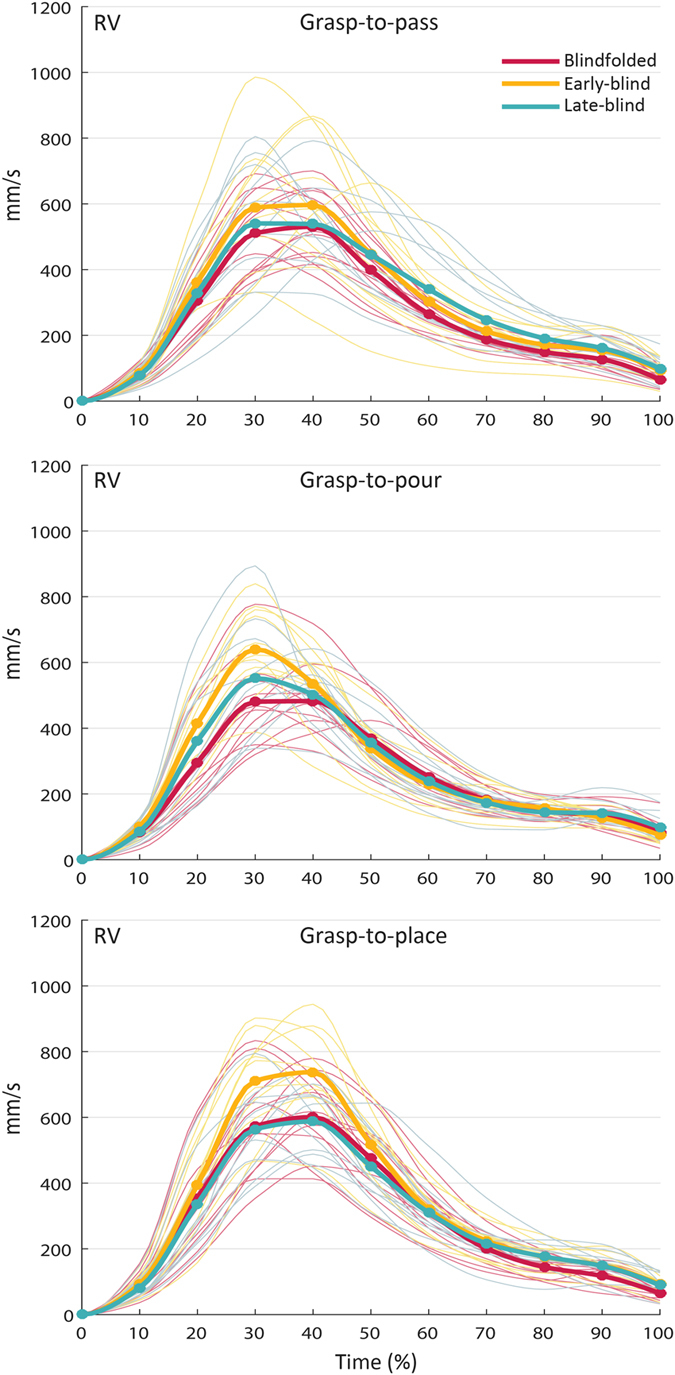
Interaction ‘visual experience’ by ‘intention’ by ‘time’ for resampled wrist velocity (RV) in early-blind, late-blind, and blindfolded sighted participants. While no influence of ‘visual experience’ on *RV* was found for grasp-to-pass movements (*upper panel*), post hoc comparisons revealed that *RV* of early-blind participants at 30% of movement time was higher compared to that of blindfolded sighted participants in grasp-to-pour movement (*central panel*). *RV* of early-blind participants at 40% of movement time was also higher compared to that of both late-blind and blindfolded sighted participants for grasp-to-place movements (*lower panel*). The bold lines represent group means; the light lines represent individual participants.

**Figure 7:**
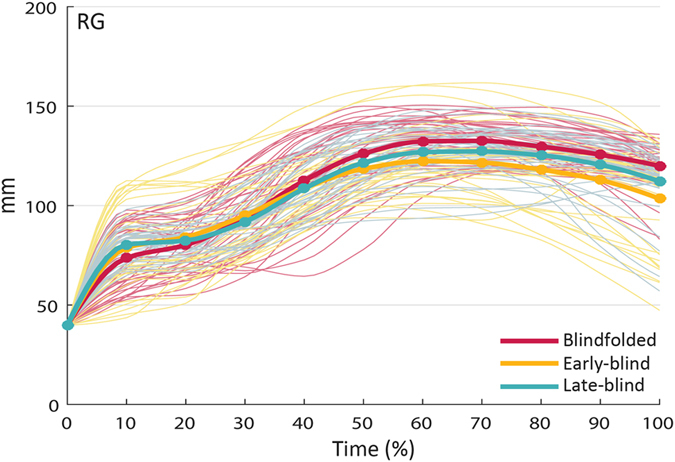
Interaction ‘visual experience’ by ‘time’ for resampled grip aperture (RG) in early-blind, late-blind, and blindfolded sighted participants. At the end of the movement (i.e., 100% of movement time), *RG* was larger for blindfolded sighted participants than for early-blind participants. The bold lines represent group means; the light lines represent individual participants.

Participants were seated on a height-adjustable chair, with their elbows and wrists resting on a table (length = 100 cm; width = 110 cm). In order to guarantee a repeatable start position, participants were asked to maintain their forearms pronated, the right arms oriented in the parasagittal plane passing through the shoulder, and their right hands in a semi-pronated position, with the tips of their thumbs and index fingers on a tape-marked point placed on the working space. A glass bottle with some water was positioned on the table at a distance of about 48 cm from participants’ midline. Depending on the experimental condition, participants were asked to reach towards and grasp the bottle with one of the following intents (Fig. 1):

1. *to pass* to pass the bottle to a co-experimenter seated in front of the participant, at approximately 100 cm distance;
2. *to pour* some water into a rectangular container (length: 18.5 cm; width: 13.0 cm; height 7.0 cm) positioned on the left side of the bottle, at a distance of 25 cm from the bottle;
3. *to place* the bottle within a cardboard box (length: 16.8 cm; width: 16.8 cm; height 12.5 cm) positioned on the left side of the bottle, at a distance of 25 cm from the bottle.

The experimenter visually monitored the performance of each trial to ensure participants’ compliance with these requirements. Early-blind and late-blind participants completed 45 movements (3 blocks of 15 movements for each intention) in a single experimental session lasting approximately 50 minutes. Sighted participants completed 45 movements in full-vision and 45 movements in no-vision (3 blocks of 15 movements for each intention for each condition). In the no-vision session, participants’ vision was blocked at all times using a blindfold. Full-vision and no-vision trials were administered in two consecutive sessions. The order of the blocks was counterbalanced across participants and sessions. For all three groups, each block was preceded by five practice trials to familiarize participants with the task. These trials were not included for data analysis.

### Kinematics recording and data processing

A near-infrared camera motion capture system with five cameras (frame rate, 100 Hz; Vicon System) was used to track the hand kinematics. Cameras were placed in the experimental room, and participants’ right hands were outfitted with 20 lightweight retro-reflective hemispheric markers (4 mm in diameter). After data collection, each trial was individually inspected for correct marker identification, and then run through a low-pass Butterworth filter with a 6 Hz cut-off. The following parameters were used for comparing reach-to-grasp movements across conditions: *movement duration* (MD), defined as the time interval (ms) between the reach onset (i.e., the first time at which the wrist velocity crossed a 20 mm/s threshold) and the grasp offset (i.e., the time at which the wrist velocity dropped below a 20 mm/s threshold); *wrist velocity* (V), defined as the velocity (mm/s) reached by the wrist marker; *wrist height* (H) defined as the z-component (mm) of the wrist marker; *wrist acceleration* (A) and *deceleration* (D), defined as the rate of change of the wrist marker velocity (mm/s^2^) with respect to time; and *grip aperture* (G), defined as the index-thumb Euclidean distance (mm). *Peak values (PV, PH, PA, PD, PG*) and *time-to-peak values*, i.e., the point in time (%) at which the respective peaks appeared (*TPV, TPH, TPA, TPD, TPG*) were extracted using a custom software (Matlab; MathWorks, Natick, MA). Additionally, to provide a better temporal characterization of the movements, *V, H*, and *G* were resampled (*R*) at intervals of 10% of the normalized movement time (from 10 to 100% of movement duration, at 10% intervals; *RV, RH, RG*).

All variables were calculated only considering the reach-to-grasp phase of the movement, from the reach onset to the grasp offset. This means that the second part of the movement, from the lift of the bottle to the completion of the action sequence, was not considered in the analysis.

### Data analysis

To investigate the influence of *visual feedback* on intention modulation, we compared the kinematics of sighted participants under full-vision and no-vision conditions. To control for family-wise error inflation, a repeated measures multivariate analysis of variance (MANOVA) was performed on *peak* and *time-to-peak* variables in order to assess the influence of ‘intention’ (3 levels; grasp-to-place, grasp-to-pass, grasp-to-pour) and ‘visual input’ (2 levels; full-vision, no-vision). An additional MANOVA was performed on *resampled* variables to assess the influence of ‘intention’ (3 levels; grasp-to-place, grasp-to-pass, grasp-to-pour), ‘visual input’ (2 levels; full-vision, no-vision), and ‘time’ (10 levels; from 10% to 100% of movement duration in 10 steps). Both MANOVAs were followed by separate ANOVAs on each of the dependent variables. Post hoc pairwise comparisons were conducted, applying the Bonferroni correction for multiple comparisons when required.

To determine the influence of *visual experience* on intention modulation, we compared the kinematics across early-blind, late-blind, and blindfolded sighted participants. The *peak* and *time-to-peak* kinematic variables were submitted to a 3 × 3 mixed MANOVA with ‘visual experience’ (early-blind, late-blind, blindfolded) as between-subject factors and ‘intention’ (grasp-to-place, grasp-to-pass, grasp-to-pour) as within-subject factor. The *R* variables were submitted to a 3 × 3 × 10 mixed MANOVA with ‘visual experience’ (early-blind, late-blind, blindfolded) as between-subject factor and ‘intention’ (grasp-to-place, grasp-to-pass, grasp-to-pour) and ‘time’ (from 10 to 100% of movement duration in 10 steps) as within-subject factors. Both MANOVAs were followed by separate ANOVAs on each of the dependent variables. Post hoc pairwise comparisons were carried out, applying the Bonferroni correction for multiple comparisons when required.

## Additional Information

**How to cite this article**: Cavallo, A. *et al*. Anticipatory action planning in blind and sighted individuals. *Sci. Rep.* **7**, 44617; doi: 10.1038/srep44617 (2017).

**Publisher's note:** Springer Nature remains neutral with regard to jurisdictional claims in published maps and institutional affiliations.

## Supplementary Material

Supplementary Information

## Figures and Tables

**Table 1:** Clinical details of all blind participants.

Participant	Gender	Age	Group	Pathology	Age of Complete Blindness	Residual Vision
001	M	49	Early-blind	Retinopathy of prematurity	Before birth	No vision
002	M	56	Early-blind	Congenital glaucoma	Before birth	No vision
003	F	32	Early-blind	Congenital cataract/Attic atrophy	Before birth	No vision
004	F	25	Early-blind	Retinopathy of prematurity	Before birth	No vision
005	F	33	Early-blind	Leber’s congenital amaurosis	Before birth	No vision
006	F	28	Early-blind	Retinopathy of prematurity	Before birth	No vision
007	M	52	Early-blind	Excess of oxygen in the incubator	Before birth	No vision
008	M	42	Early-blind	Leber’s congenital amaurosis	Before birth	No vision
009	F	26	Early-blind	Retinitis pigmentosa	At 10 years of age (before lights and shadows)	No vision
010	M	55	Early-blind	Uveitis	Before birth	Lights and shadows
011	M	45	Early-blind	Extratropia and nystagmus in retinitis pigmentosa	Before birth	No vision
012	M	27	Late-blind	Corneal opacity	At 17 years of age	No vision
013	F	49	Late-blind	Leber’s congenital amaurosis	At 46 years of age	No vision
014	M	66	Late-blind	Congenital glaucoma	At 14 years of age	No vision
015	F	22	Late-blind	Bilateral uveitis	At 12 years of age	Lights and shadows
016	M	26	Late-blind	Retinal detachment at birth	At 10 years of age	No vision
017	M	24	Late-blind	Retinis pigmentosa	At 22 years of age	No vision
018	M	42	Late-blind	Aniridia	At 18 years of age	No vision
019	M	38	Late-blind	Damage to the optic nerve	At 12 years of age	No vision
020	M	43	Late-blind	Congenital glaucoma	At 6 years of age	No vision
021	F	36	Late-blind	Retinitis pigmentosa	At 30 years of age	Lights and shadows
022	F	27	Late-blind	Retinopathy of prematurity	At 14 years of age	No vision

The table shows the gender, the age at test, the group, the pathology the age of complete blindness, and residual vision for all blind participants.
