# Supplementary material for: Anticipatory action planning in blind and sighted individuals
Source: Sci Rep. 2017 Mar 17;7:44617. doi: 10.1038/srep44617 (PMC5356336; doi:10.1038/srep44617)
Supplement: Supplementary Information [file srep44617-s1.pdf]

Anticipatory action planning in blind and sighted individuals

Andrea Cavallo[1], Caterina Ansuini[2], Monica Gori[3], Carla Tinti[1], Alessia Tonelli[3] & Cristina Becchio [1,2*]

[1] Department of Psychology, University of Turin, Italy

[2] Cognition, Motion and Neuroscience Unit, Fondazione Istituto Italiano di Tecnologia, Genova, Italy

[3] Unit for Visually Impaired People, Fondazione Istituto Italiano di Tecnologia, Genova, Italy

Supplementary Table S1. Influence of visual feedback in sighted participants. Peak and time-to-peak values (mean ± SE) for grasp-to-place, grasp-to-pass and grasp-to-pour movements under full-vision and no vision conditions.

	Grasp-to-place		Grasp-to-pass		Grasp-to-pour	
	Full Vision	No Vision	Full Vision	No Vision	Full Vision	No Vision
**Movement Duration (MD; ms)**	872.04 ± 50.63	1400.60 ± 76.12	877.94 ± 47.69	1468.97 ± 62.72	992.41 ± 61.55	1569.66 ± 64.86
**Peak Velocity (PV; mm/s)**	860.57 ± 34.20	677.80 ± 35.34	826.08 ± 28.37	592.09 ± 30.04	769.34 ± 29.56	562.26 ± 30.99
**Peak Height (PH; mm)**	181.23 ± 7.54	178.37 ± 8.91	129.68 ± 5.12	151.03 ± 9.31	134.65 ± 5.96	145.87 ± 7.71
**Peak Acceleration (PA; mm/s2)**	4005.17 ± 323.43	2870.69 ± 315.18	3797.89 ± 293.83	2394.76 ± 214.69	3269.73 ± 266.20	2296.80 ± 249.47
**Peak Deceleration (PD; mm/s2)**	-3035.07 ± 247.16	-1923.84 ± 159.32	-2788.15 ± 193.98	-1637.55 ± 134.44	-2405.88 ± 184.46	-1473.88 ± 114.45
**Peak Grip Aperture (PG; mm)**	129.64 ± 2.38	135.34 ± 3.35	132.36 ± 2.07	136.72 ± 2.72	132.92 ± 2.17	135.56 ± 2.64
**Time to Peak Velocity (TPV; %)**	44.13 ± 1.39	32.28 ± 1.50	42.82 ± 1.56	31.30 ± 0.97	41.95 ± 1.37	31.17 ± 1.54
**Time to Peak Height (TPH; %)**	71.16 ± 2.03	58.08 ± 3.86	55.38 ± 2.08	53.58 ± 3.55	55.34 ± 1.89	51.52 ± 3.40
**Time to Peak Acceleration (PA; %)**	23.39 ± 1.87	16.65 ± 0.93	23.07 ± 1.39	17.45 ± 1.16	22.57 ± 1.66	16.18 ± 1.08
**Time to Peak Deceleration (PD; %)**	66.48 ± 2.96	56.04 ± 3.81	63.96 ± 2.42	55.42 ± 3.94	62.02 ± 2.55	57.25 ± 2.90
**Time to Peak Grip Aperture (PG; %)**	77.67 ± 1.19	62.64 ± 2.67	78.31 ± 1.81	63.81 ± 2.64	79.99 ± 1.89	65.53 ± 4.03

Supplementary Table S2. Influence of visual experience in blindfolded sighted, early-blind and late-blind participants. Peak and time-to-peak values (mean ± SE) for grasp-to-place, grasp-to-pass and grasp-to-pour movements in blindfolded sighted, early-blind and late-blind participants.

	Grasp-to-place			Grasp-to-pass			Grasp-to-pour		
	**Blindfolded**	**Early-blind**	**Late-blind**	**Blindfolded**	**Early-blind**	**Late-blind**	**Blindfolded**	**Early-blind**	**Late-blind**
**Movement Duration (MD; ms)**	1400.60 ± 76.12	1301.71 ± 96.22	1345.02 ± 50.55	1468.97 ± 62.72	1478.19 ± 154.42	1296.31 ± 50.07	1569.66 ± 64.86	1625.73 ± 106.10	1600.12 ± 63.97
**Peak Velocity (PV; mm/s)**	677.80 ± 35.34	850.74 ± 42.33	681.12 ± 37.33	592.09 ± 30.03	717.18 ± 63.66	652.44 ± 47.16	562.26 ± 30.99	735.19 ± 37.44	660.13 ± 57.62
**Peak Height (PH; mm)**	178.37 ± 8.91	205.32 ± 16.21	162.47 ± 14.94	151.03 ± 9.31	161.96 ± 15.23	141.87 ± 11.19	145.87 ± 7.71	188.87 ± 17.03	154.29 ± 16.51
**Peak Acceleration (PA; mm/s2)**	2870.69 ± 315.18	3907.56 ± 316.47	3164.05 ± 204.92	2394.76 ± 214.69	3296.23 ± 351.17	2959.76 ± 280.44	2296.80 ± 249.47	3195.62 ± 255.50	3016.03 ± 405.09
**Peak Deceleration (PD; mm/s2)**	-1923.84 ± 159.32	-2846.37 ± 250.13	-1947.32 ± 162.75	-1637.55 ± 134.44	-2460.51 ± 380.09	-1905.30 ± 217.37	-1473.88 ± 114.45	-2206.56 ± 194.11	-1872.17 ± 288.43
**Peak Grip Aperture (PG; mm)**	135.37 ± 3.35	125.01 ± 4.93	131.85 ± 2.76	136.72 ± 2.72	129.79 ± 4.53	132.91 ± 3.34	135.56 ± 2.64	124.81 ± 4.29	129.93 ± 3.29
**Time to Peak Velocity (TPV; %)**	32.28 ± 1.50	31.55 ± 0.83	31.44 ± 1.54	31.30 ± 0.97	30.51 ± 1.82	32.96 ± 2.35	31.17 ± 1.54	27.43 ± 1.15	29.19 ± 1.54
**Time to Peak Height (TPH; %)**	58.08 ± 3.86	62.73 ± 5.06	58.33 ± 5.37	53.58 ± 3.55	58.19 ± 5.23	55.31 ± 4.50	51.52 ± 3.40	57.97 ± 5.33	49.81 ± 4.85
**Time to Peak Acceleration (PA; %)**	16.65 ± 0.93	18.15 ± 1.23	18.29 ± 1.92	17.44 ± 1.16	17.72 ± 1.90	17.99 ± 1.80	16.18 ± 1.08	15.40 ± 1.16	16.03 ± 1.43
**Time to Peak Deceleration (PD; %)**	56.04 ± 3.81	43.60 ± 1.20	50.77 ± 3.74	55.42 ± 3.94	53.05 ± 3.71	53.05 ± 4.50	57.25 ± 2.90	39.16 ± 1.36	46.10 ± 4.18
**Time to Peak Grip Aperture (PG; %)**	62.64 ± 2.67	59.55 ± 2.63	65.26 ± 3.76	63.81 ± 2.64	67.82 ± 2.02	71.46 ± 3.40	65.53 ± 4.03	60.85 ± 3.16	62.87 ± 4.23
